# Prokaryotic Molecular Defense Mechanisms and Their Potential Applications in Cancer Biology: A Special Consideration for Cyanobacterial Systems

**DOI:** 10.3390/cimb48010105

**Published:** 2026-01-19

**Authors:** Nermin Adel Hussein El Semary, Ahmed Fadiel, Kenneth D. Eichenbaum, Sultan A. Alhusayni

**Affiliations:** 1 Biological Sciences Department, College of Science, King Faisal University, Al-Ahsa 31982, Saudi Arabia; salhusayni@kfu.edu.sa; 2 Computational Oncology Unit, University of Chicago Medicine Comprehensive Cancer Center, 900 E 57th St, KCBD Bldg., Chicago, IL 60637, USA; fadiel01@uchicago.edu; 3 Department of Anesthesiology, Wayne State University, Detroit, MI 48341, USA; 4 Oakland University William Beaumont School of Medicine, Rochester, MI 48309, USA

**Keywords:** cancer biology, cyanobacteria, CRISPR-Cas systems, molecular defense, TALENs, ZFN

## Abstract

Cyanobacteria harbor sophisticated molecular defense systems that have evolved over billions of years to protect against viral invasion and foreign genetic elements. These ancient photosynthetic organisms possess a diverse array of restriction-modification (R-M) systems and CRISPR-Cas arrays that present challenges for genetic engineering, but also offer unique opportunities for cancer-targeted biotechnological applications. These systems exist in prokaryotes mainly as defense mechanisms but they are currently used in molecular applications as gene editing tools. Moreover, latest developments in nucleases such as zinc finger nucleases (ZFNs), TALENs (transcription-activator-like effector nucleases) are discussed. A comprehensive genomic analysis of 126 cyanobacterial species found 89% encode multiple R-M systems, averaging 3.2 systems per genome, creating formidable barriers to transformation but also providing molecular machinery that could be harnessed for precise recognition and targeting of cancer cells. This review critically examines the dual nature of these defense systems, their ecological functions, and the emerging strategies to translate their molecular precision into advanced anticancer therapeutics. Hence, the review main objectives are to explore the recent understanding of these mechanisms and to exploit the knowledge gained in opening new avenues for cancer-focused targeted interventions, while acknowledging the significant challenges to translate these systems from laboratory curiosities to practical applications.

## 1. Introduction

Cyanobacteria are one of Earth’s most significant microbial lineages, having impacted planetary atmospheric chemistry via oxygenic photosynthesis for over 2.5 billion years [1]. These ancient prokaryotes triggered the Great Oxidation Event, fundamentally altering Earth’s biosphere and enabling the evolution of complex life forms [2,3]. Today, cyanobacteria continue to impact diverse ecological niches, from marine oligotrophic environments to extreme terrestrial habitats, contributing substantially to global primary productivity and biogeochemical cycling [4,5]. Beyond their ecological roles, the sophisticated molecular defense systems of cyanobacteria offer potential tools for developing novel anticancer strategies, leveraging their precision in recognizing and responding to foreign genetic material.

There is evidence that cyanobacteria engage in quorum sensing, in which colonies can cooperate to adapt to environmental stress in a fashion that coordinates functions and is called autoinduction [6,7]. The Cyanobacterium *Gloeothece* sp. forms microcolonies of cells that are surrounded by multilaminated sheath layers which serve as a diffusion barrier to protect against toxic inhibitors [8]. This quorum sensing capability may also be linked to biofilm formation [9,10]. Kokarakis and coworkers designed quorum sensing circuits in *Synechococcus elongatus*, producing the autoinducer acyl-homoserine lactones (AHLs) as a diffusible quorum sensing circuit and coupled this to a cell elongation pathway to improve biomass harvesting [11].

The ecological success of cyanobacteria stems partly from their sophisticated cellular defense mechanisms, which have evolved to counter intense selective pressure exerted by cyanophages in aquatic environments. Marine cyanobacteria demonstrate very high documented rates of viral predation, with daily mortality rates reaching 10–50% in oceanic systems [12,13].This evolutionary proclivity facilitated the development of multiple, layered defense systems including restriction-modification complexes, CRISPR-Cas arrays [14] and various nuclease systems that collectively protect genomic integrity while regulating horizontal gene transfer and offer conceptual parallels to genome surveillance mechanisms relevant to cancer biology [15,16].

From a biotechnological perspective, these defense mechanisms present a paradox. While cyanobacteria represent plausible sustainable production platforms for biofuels, pharmaceuticals, and high-value chemicals through their ability to directly convert atmospheric CO_2_ into complex organic compounds [17,18,19]. Their endogenous nuclease systems create formidable barriers to genetic manipulation. Transformation efficiencies in cyanobacteria are often orders of magnitude lower than those of widely used model organisms such as *Escherichia coli*, which limits their development as robust synthetic biology platforms [20,21]. However, emerging research suggests that these apparent obstacles may also offer conceptual opportunities. The molecular precision with which cyanobacterial defense systems distinguish their own DNA from foreign DNA presents useful parallels to challenges in cancer biology, where selective discrimination between malignant and normal cells remains a central problem [22].

With regard to cancer or tumor formation, this is a multistep process according to [23]. The biology of cancer is rather complicated and involve environmental, genetic, and epigenetic factors. The molecular initiation of cancer is mainly by mutations that confer growth competitiveness and allow cancer progression [23]. Mutations can arise as sequence variants which originate from errors during DNA replication and repair processes, resulting from both endogenous and exogenous factors [23]. Also, mutations on chromosomal level can result from chromosomal copy number alterations and structural variations due to genomic rearrangement [23]. Identifying these mutations and removing or replacing them with normal genetic versions represent key areas of focus in cancer genome research.

Traditionally, restriction (endonucleases) enzymes were used to help diagnose cancer by identifying genetic alterations (like loss of a cut site) in tumors. Nonetheless, newer modified nucleases applications were developed to target oncogenes [24,25]. In that regard, the most recent cancer genetic targeted-therapy uses engineered nuclease enzymes like ZFNs (Zinc-Finger Nucleases) and Transcription Activator-Like Effector Nucleases (TALENs) as well as CRISPR-Cas9 to precisely cut DNA, correcting mutations or knocking out oncogenes [24,25]. These revolutionary gene-editing tools that create targeted DNA breaks for genetic modification, with the most recent CRISPR/Cas9 technology generally favored for its simplicity and higher efficiency/specificity through the use of guide RNA, while ZFNs and TALENs, regarded as earlier technologies—requiring custom protein design—offer precision but are more complex. All of these technologies work by delivering a DNA-binding domain fused to a cutting enzyme (e.g., FokI in case of ZFNs and TALENs) to specific genetic loci, relying on cell repair mechanisms [24,25]. The mechanisms and the limitations of these latest tools are going to be discussed herein.

Thus, the current review aims at shedding light on the different genetic tools that are basically based on prokaryotic nuclease enzymes essentially used for defense against viral nucleic acid invasion and genetic elements. The review explores the recent understanding of these mechanisms and ways to exploit the knowledge gained in opening new avenues for cancer-focused targeted interventions. Rather than proposing direct therapeutic deployment, this review examines how systematic understanding and rational engineering of prokaryotic defense systems in general and cyanobacteria in particular can inform cancer-related research and inspire future molecular therapeutic designs, while recognizing the substantial technical and translational barriers that need tackling The study also highlights the latest developments as well as the significant challenges to translate these systems from laboratory curiosities to practical applications. A special consideration for these systems in cyanobacteria is given.

## 2. Restriction-Modification Systems: Molecular Sentinels of Genome Integrity

### 2.1. Historical Context and Mechanistic Framework

The discovery of restriction endonucleases in the late 1960s fundamentally advanced molecular biology by enabling recombinant DNA technology and modern genetic engineering [26,27]. These enzymes function as part of restriction–modification (R–M) systems, acting as prokaryotic immune mechanisms that protect host genomes from foreign DNA through precise self/non-self-discrimination [28]. This discriminatory logic is conceptually relevant to cancer biology, where selective recognition of genomic signatures remains a central challenge for targeted therapeutic strategies.

Restriction–modification systems typically involve two complementary activities: (i) a restriction endonuclease that cleaves DNA at specific recognition sequences, and a cognate DNA methyltransferase that methylates recognition sequences in the host genome [29]. This binary system enables efficient genome protection, whereby foreign DNA lacking the appropriate methylation pattern can be rapidly degraded, while host DNA bearing the correct epigenetic modifications remains intact.

The classification of restriction enzymes into four major types reflects their distinct evolutionary origins and mechanistic approaches [30,31]. Type I systems employ large, multi-subunit complexes that translocate along DNA and cleave at variable distances from recognition sites in an ATP-dependent manner. Type II systems, which have proven highly useful in molecular cloning, cleave at or proximal to their recognition sequences and typically require only Mg^2+^ as a cofactor. Type III systems involve two inversely oriented recognition sites and introduce single-strand breaks at defined distances from these sequences. More recently described Type IV systems target pre-modified DNA, including methylated, hydroxymethylated, or glucosyl-hydroxymethylated substrates, and function as specialized counter-defense mechanisms against modified foreign genomes [31].

### 2.2. Cyanobacterial R-M System Diversity

Comparative genomic analyses demonstrated that cyanobacteria encode a wide collection of restriction-modification systems relative to other bacterial lineages. Systematic surveys of 126 completely sequenced cyanobacterial genomes found 89% have at least one R-M system and individual genomes encode an average of 3.2 systems, with certain species harboring over 20 distinct complexes [32].

This remarkable diversity (Supplementary Materials Table S1) as observed from accumulating reports (e.g., [33,34,35,36]) likely derives from intense selective pressure exerted by cyanophages in marine environments. Metagenomic studies have found that many aquatic ecosystems contain 10^7^–10^9^ virus-like particles per milliliter, with cyanophages comprising a significant component of this viral community [12,37]. The high prevalence of R-M systems in marine cyanobacteria such as *Trichodesmium erythraeum*, with 23 embedded restriction enzymes, contrasts sharply with the R-M systems in laboratory model organisms like *E. coli* K-12, which features only three systems [36,38].

Phylogenetic analysis implies that cyanobacterial R-M systems have evolved with extensive horizontal gene transfer (Table 1), with many systems having closer relationships to distantly related bacterial lineages than to systems of the same cyanobacterial species [32]. This pattern indicates ongoing evolutionary struggles between cyanobacteria and their viral predators, with R-M systems rapidly inserted, altered, and exchanged to counter evolving phage resistance mechanisms.

The methylation profiles of cyanobacterial genomes reflect this R-M system complexity. Whole-genome bisulfite sequencing studies of model organisms like *Synechocystis* sp. PCC 6803 have been found 50,000 methylated cytosine residues, primarily seen in GATC and CCWGG motifs associated with Dam and Dcm methyltransferase activities [33] and *Microcystis aeruginosa* PCC7806 [39]. However, the functional significance of these methylation sites remains unclear. Numerous modifications develop without corresponding restriction enzymes in the same genome, suggesting additional regulatory roles beyond defense functions.

### 2.3. Biotechnological Implications and Engineering Strategies

Through a synthetic biology lens, cyanobacterial R-M systems are seen to inhibit genetic manipulation. Standard transformation protocols that function efficiently in laboratory strains of *E. coli* or *B. subtilis* often show transformation efficiencies 1000–10,000-fold lower in cyanobacteria [40,41]. This limitation has held back the development of cyanobacterial platforms for biotechnological applications, despite their unique metabolic capabilities.

**Table 1:** Transformation Efficiency Improvements Through Defense System Modification.

Strategy	Organism	Specific Modification	Efficiency Improvement	Trade-Offs Observed	References
R-M system deletion	*Synechocystis* PCC 6803	Multiple R-M knockouts (GT-P strain)	10–80× improvement	UV sensitivity, reduced stress tolerance	[42]
DNA pre-methylation	*Anabaena* PCC 7120	Dam methylation of vectors	5–10× improvement	Protocol complexity	[35,43].
Restriction gene knockout	*Synechococcus* PCC 7002	hsdR deletion	2–3× improvement	Slight growth defect	[44]
Vector optimization	Multiple species	Restriction site removal	2–5× improvement	Design constraints	[45]

To circumvent barriers in order to harness these bacteria, in vitro methylation of DNA constructs using purified methyltransferases can duplicate host modification patterns and increase transformation efficiency. This approach has proven particularly successful in *Anabaena* sp. PCC 7120, where Dam methylation of incoming plasmids increased transformation efficiency by approximately 10-fold [35,43]. However, this strategy requires in-depth knowledge of species-specific methylation patterns, which is still incomplete for most cyanobacterial strains.

Alternative approaches include the systematic deletion of restriction enzyme genes to create more manageable host strains. The development of the *Synechocystis* sp. PCC 6803 GT-P strain, which lacks several R-M systems, demonstrated how targeted elimination of defense systems can significantly improve genetic tractability while maintaining basic cellular functions [42] However, such modifications often come with fitness costs, such as increased sensitivity to DNA-damaging agents and reduced phage resistance.

Vector design strategies that avoid designated sequences of known cyanobacterial restriction enzymes have also shown promise. Codon optimization algorithms can be utilized to excise restriction sites while maintaining protein function, though this approach is limited by incomplete knowledge of the full spectrum of cyanobacterial R-M sequence selectivity [45] Additional approaches combine multiple strategies, including host modification, vector optimization, and specialized transformation protocols.

### 2.4. Emerging Applications in Precision Medicine

Recent advances in understanding cyanobacterial R-M systems have shed light on the specificity with which these systems recognize DNA methylation patterns. This can impact precision therapeutic approaches, as seen in oncology where DNA methylation abnormalities are hallmarks of malignant transformation [46].

Cancer cells exhibit distinct DNA methylation profiles relative to normal tissue, with widespread hypomethylation of repeated elements and localized hypermethylation of CpG islands in tumor suppressor gene promoters [47]. This epigenetic dysregulation provides the opportunity for selective targeting using customizable restriction systems that recognize cancer-specific methylation patterns. Preliminary studies identified restriction enzymes that can be designed to adapt to tumor-associated DNA modifications, providing a strategy for the destruction of malignant cells while sparing normal tissue [48]. The integration of quorum sensing circuit designs that can include positive and negative feedback loops along with promoters can provide an opportunity to develop auto-induction systems to enhance defensive targeting.

Biocontainment properties inherent to R-M systems make them attractive for therapeutic applications requiring strict localization. Engineered cyanobacteria carrying tumor-responsive restriction circuits could likely survive only within specific microenvironments characterized by DNA methylation landscapes, providing an additional safety mechanism for live therapeutic microorganisms [49].

#### 2.4.1. Genome Editing

The idea of genome editing is based on the use of chimeric nucleases composed of sequence-specific DNA-binding domains fused to a non-specific DNA cleavage nuclease. They enable efficient and precise genetic modifications in different organisms [24]. ZFNs (Zinc Finger Nucleases) and TALENs (Transcription Activator-Like Effector Nucleases) are programmable protein tools for targeted gene editing, creating DNA double-strand breaks at specific sites to enable gene knockout or insertion by leveraging the cell’s repair mechanisms (NHEJ/HR). ZFNs use custom zinc finger proteins for DNA binding, while TALENs use TALE protein repeats, both fused to the FokI nuclease domain, with TALENs generally considered easier to engineer due to their simpler, modular DNA-binding code.

#### 2.4.2. A-TALENs

A restriction endonuclease called Fok1 was found to naturally occur in *Flavobacterium okeanokoite.* This restriction endonuclease is a bacterial type IIS. It consists of two domains; DNA-binding domain at the N-terminal and a non sequence-specific DNA cleavage domain at the C-terminal. Once the protein is bound to DNA at the 5’-GGATG-3’ recognition site, the DNA cleavage domain, cleaves the DNA at two locations, regardless of the nucleotide sequence [50] producing sticky ends. Fusion of transcription activator-like (TAL) proteins and a nuclease (FokI) occurs in the following manner TAL proteins contain amino acid repeating motifs with variable positions which can recognize specific nucleotides. When fused to nucleases, DNA binding domains can be used to induce double stranded breaks. The mechanism of action employs a pair of chimeric proteins, each composed of a TAL effector DNA-binding domain (recognizing a specific sequence) fused to a FokI nuclease domain The pair of proteins are designed to bind to a pair of target sites in the genome, each ~18 bp long and flanking a 14–20 bp spacer. Upon binding to DNA, the Fokl nuclease domains on the pair of proteins are able to dimerize, which in turn leads to DNA cleavage within the spacer region between the two target sites [24] and (https://en.vectorbuilder.com/resources/faq/crispr-vs-talen.html#:~:text=First%2C%20for%20vector%20construction%2C%20CRISPR,for%20each%20protein%2DDNA%20interaction, accessed on 3 January 2026).

#### 2.4.3. B-ZENs

These are specifically designed zinc finger nucleases that identify 3 bp DNA sequences, equipped with the FokI nuclease domain [51]. ZFNs cut DNA at a specific location based on the arrangement of ZFPs that are designed to precisely identify the selected target site within a genome. The action mechanism involves the attachment of the two ZFNs to neighboring DNA locations, allowing FokI to dimerize and cleave. The (Cys2His2) ZFPs is arguably the optimal design for creating tailored ZFN molecules with novel sequence-specificities. It is regarded as one of the initial genetic tools that provided high specificity, yet it remained complicated to construct and less effective than CRISPR, which will be addressed later.

ZFNs and TAlENs share characteristics, such having a DNA-binding domain (ZF or TALE) fused with the FokI nuclease which undergoes a dimerization stage. In this stage, two monomers attach to opposite DNA strands, facilitating the assembly of FokI domains to cut the DNA. They both induce double strand breaks. Nonetheless, there are distinctions as well, particularly in their DNA recognition and binding mechanisms. ZFNs utilize engineered zinc finger proteins, which can be quite complex to design, to identify DNA triplets (3-base pairs). TALENs, conversely, utilizes TALE protein repeats, with each repeat identifying a specific base pair (1:1 code). This modularity simplifies their design and construction for new targets. Another distinction lies in their source, as ZFNs are derived from naturally occurring zinc finger proteins. On the other hand, TALENs originate from TALE proteins released by *Xanthomonas* bacteria. Another key difference is the user-friendliness: TALENs typically provide increased flexibility and easier design compared to ZFNs, making them more approachable for scientists.

#### 2.4.4. Genome Editing of Oncogenes Using ZFNs and TALENs

Genome editing of oncogenes using Zinc-finger nucleases (ZFNs) and transcription activator-like effector nucleases (TALENs) involves significant design challenges that can limit their effectiveness and safety in cancer research and therapy according to [52].

#### 2.4.5. Key Caveats in Nuclease Design

− Targeting Complexity and Context Dependency (ZFNs): ZFNs are composed of modules that each recognize a 3-base pair (bp) sequence. A major caveat is that the binding of one zinc finger often influences neighboring modules, making modular assembly difficult and frequently leading to poor binding efficiency or off-target effects.
− Design Constraints (TALENs): While TALENs are more modular than ZFNs due to a one-to-one correspondence between TALE repeats and single nucleotides, they still face restrictions. For instance, conventional TALENs require a thymine (T) at the 5′ end of the target sequence.
− Off-Target Effects and Cytotoxicity: Both technologies risk inducing double-strand breaks (at unintended genomic sites, which can lead to deleterious mutations or cytotoxicity. ZFNs generally exhibit higher off-target rates and greater toxicity than TALENs.
− Delivery Bottlenecks: The large size of TALENs (~3 kb per monomer) makes them difficult to package into viral vectors like adeno-associated virus (AAV), which has a capacity of ~5 kb. ZFNs are smaller (~1 kb) and more compatible with various delivery systems.
− Low Editing Efficiency in Malignant Cells: Studies targeting specific oncogenes have shown that nucleases with high activity in simple assays (like yeast-based models) may yield negligible activity in tumor cell lines, often due to improper binding or chromatin accessibility issues.

Nonetheless, [53] reviewed several successful experiments conducted on oncogenes editing using ZFN and TALEN including one study that used custom-designed ZFN for a human lymphoblast cell from chronic myeloid leukemia patient. Lymphocytes treated with ZFNs lacked the surface expression of CD3-TCR [54].

## 3. CRISPR-Cas Systems: Adaptive Immunity and Programmable Genome Editing

### 3.1. Discovery and Mechanistic Understanding

Prokaryotes were found to possess an RNA-guided immune system, characterized by CRISPR loci and CRISPR-associated genes that grant adaptive immunity against bacteriophage infections [55]. During the immunization step, short segments of invading foreign DNA are integrated into the host chromosome’s CRISPR repeat spacer as new spacers. CRISPR-Cas systems operate through a highly evolved three-stage process: 1—adaptation (spacer acquisition), 2—the expression and 3—processing of CRISPR RNA, and interference with target nucleic acids [55]. During the adaptation phase, Cas proteins capture fragments of foreign DNA and integrate them as spacers that are placed between identical repeated sequences in CRISPR arrays [56]. These spacers serve as molecular memories of past infections, providing heritable evidence of encountered genetic elements. The expression phase involves transcribing CRISPR arrays into long precursor RNA molecules that are later processed by Cas endonucleases into individual guide RNAs. Finally, during interference, guide RNAs lead Cas protein complexes to complementary sequences in foreign nucleic acids, which prompts targeted degradation.

The discovery that CRISPR-Cas systems could be programmed with synthetic guide RNAs to target specific genomic loci upgraded these natural defense mechanisms into versatile genome-editing tools [57,58].

#### Mechanism of Action of CRISPR/Cas9 in Gene-Editing

A short guide RNA (gRNA) is employed to steer the Cas9 enzyme towards a specific DNA sequence. The gRNA directs Cas9 to snip the DNA. To accurately modify a particular genetic locus, a donor vector for homologous recombination or a long oligo featuring the desired edit sequence surrounded by the adjacent upstream and downstream homology arms of the target site can be introduced into the cells along with gRNA(s) and Cas9. This method is significantly easier and quicker to develop (just modify the gRNA), frequently more efficient and precise, and enables concurrent editing of several genes by necessitating a Protospacer Adjacent Motif (PAM). This breakthrough has revolutionized molecular biology and opened new possibilities for therapeutic applications across diverse fields, from genetic disease treatment to cancer therapy. Additional tools such as riboswitch-inducible CRISPR/Cas systems for precise and markerless editing have been studied in *Synechostcystis* [59].

CRISPR-Cas systems were first discovered in cyanobacteria, though their function remained enigmatic for nearly two decades following their initial observation [60,61]. The realization that clustered regularly interspaced short palindromic repeats functioned as adaptive immune systems has become one of the most significant discoveries in microbiology, fundamentally enhancing our understanding of prokaryotic defense mechanisms [56,62].

Use of CRISPR/Cas9 technique in cancer therapy was successful in bran cancer where the technique was used to introducing a Knock-out of Nf1, Pten, and Trp53 genes which are responsible for glioblastoma and Ptch1 gene responsible for [63]. With regard to colorectal cancer, Ref. [64] reported that several oncogenes were mutated. With regard to hepatocarcinoma, Qi et al., 2020 [65] used a lactose-derived CRISPR/Cas9 delivery system for efficient genome editing in vivo to treat hepatocellular carcinoma.

### 3.2. Cyanobacterial CRISPR Diversity and Function

Cyanobacteria exhibit remarkable diversity in their CRISPR-Cas systems, with current surveys identifying functional systems in approximately 37% of sequenced genomes [66]. This prevalence reflects the intense selective pressure exerted by cyanophages in aquatic environments, where CRISPR-mediated immunity provides crucial protection against viral predation.

The classification of CRISPR-Cas systems into two major classes and six types reveals the evolving complexity of these defense mechanisms [67]. Class 1 systems employ multi-protein effector complexes to process RNA and target interference, while Class 2 systems generate single, large proteins for these functions. Within cyanobacteria, Type I systems are common, with I-D and I-E subtypes found most frequently. The Type I-E system observed in *Synechocystis* sp. PCC 6803 has been particularly well-characterized, with its multi-subunit Cascade complex that processes CRISPR RNA and a distinct Cas3 nuclease that degrades target DNA [68].

Analysis of spacer content in cyanobacterial CRISPR arrays identifies the ecological pressures shaping these systems (Table 2). Many arrays contain spacers targeting plasmids and transposable elements distinct from viruses, suggesting regulation of horizontal gene transfer and maintenance of genome stability [66,69]. Rapid turnover of spacers in natural populations indicates ongoing competition between cyanobacteria and their genetic parasites, with new spacers being continuously acquired to counter evolving threats.

Some cyanobacterial CRISPR systems appear to target sequences within their own genomes, potentially providing oversight rather than defensive functions [68]. This self-targeting suggests that CRISPR-Cas systems may have evolved additional roles outside of immunity, including gene regulation and genome maintenance. However, mechanisms preventing self-destruction in such systems remain poorly understood and represent an active area of investigation. Nonetheless, there are several reports on CRISPR-Cas applications in cyanobacterial research as found in Table 3.

### 3.3. Biotechnological Applications and Challenges

The adaptation of CRISPR-Cas systems for cyanobacterial genome editing has transformed synthetic biology research in these organisms. Since the initial validation in 2013, CRISPR-based tools have enabled precise genetic modifications that were previously difficult or impossible to achieve [74,78]. Applications range from simple gene knockouts to complex metabolic pathway engineering, with success rates varying dramatically depending on the target organism and specific modifications attempted.

One of the most significant advantages of CRISPR technology in cyanobacteria is its ability to overcome certain limitations found with polyploidy. Many cyanobacterial species maintain multiple chromosome copies, complicating traditional genetic manipulation that relies on complete gene replacement [79]. CRISPR interference (CRISPRi) using catalytically dead Cas proteins can silence targeted genes without requiring editing of each chromosome copy, thereby allowing functional studies and metabolic engineering in polyploid organisms [75,80]

However, several challenges continue to limit the widespread application of CRISPR technology in cyanobacteria. Delivery of CRISPR components remains difficult, as transformation efficiency is variable between species and many strains remain genetically intractable [21]. The toxicity associated with Cas protein expression in some cyanobacterial hosts also impedes optimization, potentially giving rise to off-target DNA binding or interference with essential cellular processes [70].

Protospacer adjacent motif (PAM) requirements also complicate targeting options, particularly in AT-rich cyanobacterial genomes where usable PAM sequences may be limited near desired target sites [57]. The development of engineered Cas proteins with relaxed PAM requirements and the presence of natural Cas variants with different PAM specificities have begun to address these limitations [81,82].

### 3.4. Therapeutic Potential and Cancer Applications

The precision and programmability of CRISPR-Cas systems for cancer treatment allows genome editing to address fundamental molecular drivers of malignancy [83]. Targeting specific oncogenes, tumor suppressor genes, or regulatory elements with high specificity offers unprecedented opportunities for precision medicine approaches.

Several strategies for CRISPR-mediated cancer therapy are currently under investigation. Direct targeting of oncogenic mutations using Cas9, or other effector proteins could potentially restore normal cellular activities by fixing or blocking driver mutations [84]. Base editing and prime editing technologies enable more subtle modifications, allowing correction of point mutations in the absence of double-strand DNA breaks [85]. Cyanobacteria have also been employed to enhance tumor targeting in photodynamic therapy [86]. Moreover, it was reported that genome-editing by unidirectional deletion in human T cells using type I-B system from *Synechocystis* sp. PCC 6714 proved successful [87].

CRISPR-based immunotherapy approaches using engineered T cells carrying CRISPR systems presage enhanced antitumor activity in preclinical models [88]. The ability to precisely edit immune cell genomes may overcome many limitations of current immunotherapy approaches such as immune evasion and systemic toxicity.

However, significant challenges remain in translating CRISPR technology to clinical cancer applications. Off-target editing presents safety concerns, particularly for heritable modifications, while isolating specific cell types remains technically challenging [89]. Manufacturing and quality control for CRISPR therapeutics also require curated approaches to ensure consistency and safety. Also, the use of the system should not be exclusive to mammalian cells. Other biological entities such as plants can benefit from the technology especially those that are with potent anticancer properties as discussed below.

## 4. The Cyanobacterial Defense Paradox: Barriers as Opportunities

### 4.1. Systems-Level Interactions and Evolutionary Trade-Offs

The coexistence of multiple defense systems within individual cyanobacterial genomes creates complex molecular networks with both synergistic and competitive interactions. Recent research has revealed that R-M systems and CRISPR-Cas arrays can work cooperatively, where restriction enzymes develop DNA fragments that seed CRISPR spacer acquisition [90,91]. This interaction creates a hierarchical defense strategy in which immediate restriction-based immunity is augmented by adaptive CRISPR memory formation.

However, conserving multiple defense systems also involves evolutionary trade-offs. Each system requires metabolic resources for protein synthesis and maintenance, and retaining numerous nucleases can create cellular stress or inhibit essential processes [92]. The observation that laboratory strains often lose their defense systems during extended cultivation suggests that these systems exact fitness costs in the absence of selective pressure from genetic parasites.

The complexity of cyanobacterial defense networks can sometimes result in exploited system weaknesses. Phages have evolved sophisticated mechanisms to overcome both R-M and CRISPR defenses, including anti-restriction proteins, CRISPR inhibitors, and rapid sequence evolution that avoid recognition [93,94]. This ongoing evolutionary arms race drives continuous innovation in both attack and defense mechanisms, creating dynamic molecular adaptation that challenge simple engineering approaches. A comparative defense System analysis in between two cyanobacteria and *E. coli* found in Supplementary Materials Table S2. Shows that CRISPR machinery in cyanobacterium *Synechocystis* sp. outperforms that of *E. coli* indicating the excellent potentials of this cyanobacterium in genome editing applications. This is only the beginning as more cyanobacterial genomes are deciphered and more information on molecular defense tools are revealed continuously.

### 4.2. Biocontainment and Safety Considerations

One of the most promising aspects of cyanobacterial defense systems for biotechnological applications is in their potential for biocontainment. Reliance on specific sequential molecular signals creates opportunities for designing organisms that can survive only under defined conditions, addressing safety and toxicity concerns about environmental release of genetically modified organisms [49].

Engineered restriction circuits that act based on synthetic molecules or specific environmental conditions could function as molecular kill switches, preventing modified organisms from persisting outside intended applications [95]. Similarly, CRISPR systems could be programmed to target essential genes based on specific signals, providing additional layers of biocontainment.

The development of such biocontainment systems requires careful consideration of guardrails for potential failure modes and evolutionary pressures that might select for escape variants. Redundant safety mechanisms, rigorous testing protocols, and ongoing monitoring systems are essential components of any strategy for environmental deployment of engineered cyanobacteria.

### 4.3. Regulatory and Ethical Considerations

The dual-use nature of cyanobacterial defense systems raises important regulatory and ethical questions. While these systems offer potential for beneficial applications, they could also be misused for harmful purposes. The ability to program nucleases for specific targeting capabilities requires careful oversight and appropriate safety measures.

Regulatory frameworks for biotechnology applications must balance innovation and safety, particularly for therapeutic applications where direct human exposure is planned. Standardized safety protocols, quality control, and post-market surveillance systems are paramount for the responsible development of these technologies [96].

International coordination may be necessary to enforce consistent safety standards and prevent regulatory arbitrage that could compromise global biosafety. Professional societies and scientific research organizations have important roles for developing best practices and ethical guidelines related to the application of these powerful technologies.

## 5. Future Perspectives and Technological Horizons

### 5.1. Advancing Fundamental Understanding

Despite significant progress in characterizing cyanobacterial defense systems, major knowledge gaps still limit designing practical applications. Systematic functional characterization of the thousands of predicted restriction enzymes and CRISPR systems found in cyanobacterial genomes represents a massive undertaking that will require coordinated international efforts.

High-throughput approaches combining computational prediction, automated cloning, and robotic screening platforms could accelerate the pace of discovery and characterization. Long-read sequencing technologies permit complete genome assemblies that describe defense system organization, while single-molecule methylation detection helps map epigenetic landscapes [97,98].

Structural biology approaches using cryo-electron microscopy and X-ray crystallography continue to provide mechanistic insights for protein engineering [99]. Understanding molecular specificity, control of allosteric binding, and protein–protein interactions facilitate rational design of modified systems with improved properties.

### 5.2. Synthetic Biology Applications

The integration of cyanobacterial defense systems into synthetic biology platforms requires development of standardized tools, protocols, and design principles. BioBrick-style registries of characterized components may improve system assembly and enable predictable engineering outcomes [100]. Computational tools for designing guide RNAs, anticipating off-target effects, and optimizing expression are essential infrastructure for routine application.

Standardized chassis organisms with well-characterized defense system profiles could provide reliable platforms for biotechnological applications. Designing model strains that balance genetic tractability with ecological robustness is an important component for development [45].

Advanced applications may require the development of entirely synthetic defense circuits that combine components from different organisms or incorporate novel functionalities. Such hybrid systems could provide capabilities not found in nature while maintaining the specificity and reliability of evolved systems.

### 5.3. Clinical Translation Pathways

The path from laboratory discovery to clinical application requires addressing numerous technical, regulatory, and economic challenges. In that regard, [53] outlined the clinical application by two stages: ex vivo and in vivo genome editing for clinical therapy. For ex vivo, cells are isolated from a patient, edited and then reintroduced back to the patient. It is important to notice that to achieve therapeutic success, the target cells must be able to survive in vitro and return to the target tissue after transplantation. With regard to in vivo editing, engineered nucleases are to be delivered by viral or non-viral route and then directly injected into the patient. Suitable delivery method/vector together with manufacturing scalability remain significant obstacles, as current transformation and cultivation protocols are not well-suited to industrial-scale production especially for engineered cyanobacteria.

Clinical trial design for cyanobacterial therapeutics will require specialized approaches with safety protocols that account for the unique properties of these organisms. Efficacy assessment may require novel biomarkers and endpoints that capture the complex interactions between engineered organisms and human physiology.

Economic considerations include patent landscape navigation, manufacturing cost optimization, and market access strategies. The development of sustainable business models that support continued research and development while ensuring patient access remains a challenge for the biotechnology industry.

### 5.4. Potential Use in Medical Plants as Illustative Example of the Applications of Crispr Technology

Latest report on herbal medicine estimates that more than 75% of the world population uses herbal medicine to treat a wide range of diseases, from seasonal bacterial infections to chronic conditions [101]. In vitro and in vivo studies on bioactive compounds from commonly used medicinal plants have shown preventive and therapeutic effects against many diseases, including cancer. For instance, tanshinone IIA, a major diterpenoid compound found in *Salvia miltiorrhiza* plant species, exhibits anticancer activity in numerous cancer cell types originating from bone, lung, skin, stomach, colon, ovary, breast, and prostate [102,103,104,105,106,107,108,109,110]. Likewise, research on withanolides, bioactive steroidal lactones produced by several species of the *Withania* genus, have demonstrated their potent anticancer properties through apoptosis induction in multiple human cancer cell lines [111,112,113,114].

However, the full therapeutic exploitation of these compounds is limited because their natural abundance in producing plants is often low [115]. CRISPR–Cas technology offers a promising solution for enhancing plant bioactive-compound production by engineering or boosting targeted biosynthetic pathways. A notable example is the CRISPR/Cas9-mediated mutation of the upstream open reading frame (uORF), a translational suppressor, in the diterpene synthase (*SmCPS1*) gene of the tanshinone biosynthetic pathway in *S. miltiorrhiza*, which significantly increased SmCPS1 protein content and consequently elevated tanshinone accumulation. Similarly, the use of a CRISPR/Cas9 multiplex-knockout system to simultaneously disrupt the *SmCPS3* and *SmCPS4* genes, which encode enzymes that divert metabolic flux away from tanshinone biosynthesis, further shifted precursor flow toward tanshinone production [116,117]. Thus, CRISPR-based metabolic engineering offers a sustainable and scalable approach for increasing the production of plant-derived anticancer compounds for future therapeutic use.

## 6. Discussion

With regard to cancer therapy, quite recently genome-editing using CRISPR-Cas was successful in human T cells using type I-B system from *Synechocystis* sp. PCC 6714 [87]. They made unidirectional 4.5 kb deletion in TRAC locus with an editing efficiency up to 41.2%. These results are promising; however, the technology is still progressing and certain challenges need to be tackled. For example, it is reported that the accumulation of Cas9 is toxic to cyanobacteria; therefore, the finetuning of the expression of Cas protein needs to be established [74,118]. A theophylline-responsive riboswitch was developed to tightly regulate RNA device in order to keep Cas9 at level low enough to prevent its toxicity and high enough to induce genome editing [59] In addition, this circuit could be assembled into a shuttle vector to avoid labor and time-consuming genetic integration [119]. With regard to cyanobacterial CRISPR system potential, A comparative defense System analysis between *Synechocystis* sp cyanobacterium and *E. coli* showed the greater cyanobacterial potentials that await exploitation.

When comparing CRISPR to TALEN as genome editing tools, we find that both are used to knock out genes, or to knock in point mutations or insertions, but these two systems are different in several aspects [25,120] Some of these aspects are:

− Ease of Use: CRISPR is easiest (RNA-based), TALENs are intermediate (protein-based), ZFNs are most complex (protein-based).
− Efficiency/Specificity: CRISPR generally outperforms ZFNs and TALENs in efficiency and often specificity, though all can have off-target effects [121]. Multiple gene mutations can be introduced concurrently with a single injection in case of CRISPR, whereas TALENs are limited to simple mutations. CRISPR transfections also have a higher efficiency, whereas TALEN editing often results in mosaicism, i.e., some but not all cells are edited [122].
− Generations: ZFNs and TALENs paved the way [53], with CRISPR/Cas9 emerging as the dominant, versatile, and powerful third-generation gene-editing tools.

Nonetheless, it is also important to note that TALEN is generated to target nearly any sequence in the genome. On the other hand, CRISPR is limited by the requirement for a PAM sequence (NGG) located on 3′ end of guide RNA target sequence. Knocking out genes is largely because cleavage anywhere in the gene is potentially effective, but may present difficulties in generating site-specific mutations or insertions that require cleavage at a specific position of the gene. On the other hand, CRISPR outcompetes TALEN in several ways. First, for vector construction, CRISPR system only needs to construct a short gRNA because targeting of Cas9/gRNA complex relies on simple RNA/DNA hybridization, while TALEN system requires re-engineering of the TAL DNA-binding domain that is unique for each protein-DNA interaction. Therefore, gRNAs are cheaper and easier to design and construct than TALENs which always require two vectors per target site. Secondly, for some applications, such as injecting mouse embryos, Cas9 protein and gRNA can be more efficiently delivered via direct injection, but TALEN cannot. Thirdly, CRISPR is extremely versatile in genetic screening experiments since CRISPR screening library expressing many thousands different gRNAs can be easily constructed in a high-throughput manner. The major limitation to CRISPR-Cas technology in general in cancer therapy according to [121] is that edited cells are usually less fit compared to unedited cells, hence their therapeutic effects is reduced. Another limitation is the delivery technique as well as off-target impacts. Delivery vectors of Cas9/sgRNA, include viruses, plasmids and nanoparticles. as well as electroporation, microinjections, and lipid-mediated transfection. According to [53] and references therein, there are three common strategies for genome editing using CRISPR/Cas9; (1) using a plasmid to encode Cas9 protein and sgRNA, However, the recombinant plasmid needs to be introduced into the nucleus of target cells, which is a challenge; (2) direct intracellular delivery of Cas9 messenger RNA (mRNA) and sgRNA, but poor stability of mRNA can results in transient expression of mRNA and a short duration of gene modification; (3) directly delivery of Cas9 protein and sgRNA which ensures rapid action, stability, and limited antigenicity.

In cyanobacteria, several challenges limit the widespread application of CRISPR technology as the Delivery of CRISPR components that remains difficult, and the limited rate of success of transformation efficiency and many strains remain genetically intractable [21]. The toxicity associated with Cas protein expression in some cyanobacterial hosts is another obstacle [70] Protospacer adjacent motif (PAM) requirements also complicate targeting options, particularly in AT-rich cyanobacterial genomes where usable PAM sequences may be limited near desired target sites [57].

## 7. Conclusions

Cyanobacterial restriction-modification systems and CRISPR-Cas arrays are remarkable evolutionary innovations that have protected these ancient organisms for billions of years. Their molecular sophistication, evolved through countless cycles of selection and counter-selection, provides both inspiration and practical tools for contemporary biotechnology applications.

The transformation of these systems from biotechnological obstacles to therapeutic opportunities exemplifies how fundamental biological research can yield unexpected practical benefits. By systematically characterizing, understanding, and engineering cyanobacterial defense mechanisms, researchers are developing new approaches to longstanding challenges in medicine and biotechnology.

Technical challenges in genetic manipulation, delivery, and safety and regulatory frameworks require oversight and standards. Economic and social factors will ultimately determine which applications achieve widespread implementation. The interdisciplinary nature of this field requires collaboration between microbiologists, synthetic biologists, clinicians, and engineers.

As we face growing global challenges in health, sustainability, and resource management, the biological wisdom encoded in current and newly engineered cyanobacterial genomes offers valuable tools for developing solutions. The careful, responsible development of these ancient defense systems may provide keys to developing technologies that are simultaneously more precise, more sustainable, and more effective than conventional approaches.

## Figures and Tables

**Table 2:** CRISPR-Cas System Distribution in Characterized Cyanobacterial Strains.

Organism	CRISPR Arrays	Cas Type	Total Spacers	Activity Status	References
*Synechocystis* sp. PCC 6803	3	I-E	47	Experimentally confirmed	[68]
*Synechococcus* sp. PCC 7002	2	I-D	23	Active (inferred)	[70]
*Anabaena* sp. PCC 7120	5	I-E, III-A	89	Partial activity	[71]
*Nostoc punctiforme* ATCC 29133	7	I-E, I-D	156	Mixed activity	[72]

Note: Activity status based on Cas-gene expression evidence and spacer acquisition patterns from published studies. Selected data in Table were discussed in [73].

**Table 3:** Documented CRISPR-Cas Applications in Cyanobacterial Research.

Application	Organism	Target Genes	Success Rate *	Product/Phenotype	References
Gene knockout	*Synechocystis* PCC 6803	*slr0168*	15–25%	Metabolic modification	[74]
CRISPRi knockdown	*Synechocystis* PCC 6803	*glgA*, *glgC*	70–95%	Glycogen reduction	[75]
Multiplex editing	*Synechococcus* PCC 7002	Multiple targets	10–30%	Pathway modification	[76]

* Success rates refer to percentage of transformants with intended genotype modifications. Selected data in Table were discussed in [77]

## Data Availability

No new data were generated in this study.

## Supplementary Materials

The following supporting information can be downloaded at: https://www.mdpi.com/article/10.3390/cimb48010105/s1; Table S1: Restriction-Modification System Diversity in Selected Cyanobacterial Genomes. Table S2: Comparative Defense System Analysis in Selected Organisms. References [33,35,36,68,70,123,124,125] have been cited in the Supplementary Materials.
