# Supplementary material for: Prokaryotic Molecular Defense Mechanisms and Their Potential Applications in Cancer Biology: A Special Consideration for Cyanobacterial Systems
Source: Curr Issues Mol Biol. 2026 Jan 19;48(1):105. doi: 10.3390/cimb48010105 (PMC12840210; doi:10.3390/cimb48010105)
Supplement: Supplementary file 1 [file cimb-48-00105-s001.zip › cimb-4064660-supplementary.pdf]

Table S1. Restriction-Modification System Diversity in Selected Cyanobacterial Genomes.

**Organism**	**Genome Size (Mb)**	**Predicted R-M Systems**	**Type II Systems**	**Methyltransferases**	**Transformation Efficiency**	**References**
Synechocystis sp. PCC 6803	3.57	8-12*	6-8	12-15	10^3-10^4 CFU/μg	Matveyev et al., 2001
Synechococcus sp. PCC 7002	3.01	10-15*	8-11	15-18	10^4-10^5 CFU/μg	Ungerer & Pakrasi, 2016
Anabaena sp. PCC 7120	7.21	15-22*	10-14	20-28	10^2-10^3 CFU/μg	Elhai & Wolk, 1988
E. coli K-12 (control)	4.64	3	3	3	10^8-10^9 CFU/μg	Blattner et al., 1997

*Numbers represent ranges reported across different studies due to annotation variations and computational prediction uncertainties.

Table S2: Comparative Defense System Analysis in Selected Organisms

Parameter	*Synechocystis* PCC 6803	*Anabaena* PCC 7120	*E. coli* K-12
CRISPR-Cas systems	Present: Type I-D plus two Type III systems (on plasmid pSYSA) (Scholz et al 2013)	Not well documented / system unclear in PCC 7120 (less evidence)	No fully functional native CRISPR-Cas system in many K-12 lab strains (arrays present but cas genes absent or inactive)
CRISPR arrays	Three arrays on pSYSA plasmid (*Synechocystis*) (Ikeuchi and Tabata, 2001)	Data insufficient / "not established"	Two arrays in K-12, but no cas genes (inactive)
Restriction–modification systems	*Synechocystis*: multiple methyltransferases documented; restriction endonuclease activity historically absent or weak in early reports (Huang F et al., 2002)	*Anabaena*: some R-M genes annotated, but detailed functional data limited	*E. coli* K-12: well-characterized R-M systems (EcoKI, EcoB etc)
DNA methylation motifs	*Synechocystis*: documented many methyltransferases; exact motif counts vary; GC content ~47.7% (Kaneko and Tabata, 1997)	*Anabaena*: GC content and methylome less thoroughly published	E. coli K-12: Dam (GATC), Dcm (CCWGG) widely cited
Natural transformation	Yes	Not reliably (see above)	No (requires artificial methods)
GC content	~47.7% (*Synechocystis*) (Kaneko and Tabata, 1997)	~41% (*Anabaena*) (not included above but approximate)	~50–51% for E. coli K-12
